# Coconut Residue-Derived Nanoporous Carbon via Hydrothermal Carbonization for Nanoporous Carbon-Based Supercapacitor Electrodes

**DOI:** 10.3390/polym17131752

**Published:** 2025-06-25

**Authors:** Kemchat Ruenroengrit, Jumpon Kunyuan, Nuttapong Ruttanadech, Napat Kaewtrakulchai, Pramote Puengjinda, Nattapat Chaiammart, Sutee Chutipaijit, Achanai Buasri, Masayoshi Fuji, Apiluck Eiad-Ua, Gasidit Panomsuwan

**Affiliations:** 1 Nanoscience and Nanotechnology, College of Materials Innovation and Technology, King Mongkut’s Institute of Technology Ladkrabang, Ladkrabang, Bangkok 10520, Thailand; 65116002@kmitl.ac.th (K.R.); sutee.ch@kmitl.ac.th (S.C.); 2 King Mongkut’s Institute of Technology Ladkrabang Prince of Chumphon Campus, Chumphon 86160, Thailand; jumpon.ku@kmitl.ac.th (J.K.); nuttapong.ru@kmitl.ac.th (N.R.); 3 Kasetsart Agricultural and Agro-Industrial Product Improvement Institute, Kasetsart University, Bangkok 10900, Thailand; knapat.kara@gmail.com; 4 Deutsche Gesellschaft für Internationale Zusammenarbeit (GIZ) GmbH, Bangkok 10110, Thailand; pramote.puengjinda@giz.de; 5 Department of Materials Science and Engineering, Faculty of Engineering and Industrial Technology, Silpakorn University, Nakhon Pathom 73000, Thailand; nattaphat.c@ku.th (N.C.); achanai130@gmail.com (A.B.); 6 Advanced Ceramic Center, Nagoya Institute of Technology, Gifu 466-8555, Japan; fuji@nitech.ac.jp; 7 Department of Materials Engineering, Faculty of Engineering, Kasetsart University, Chatuchak, Bangkok 10900, Thailand; gasidit.p@ku.ac.th

**Keywords:** coconut residue, hydrothermal carbonization, KOH activation, nanoporous carbon, supercapacitor electrodes

## Abstract

The increasing demand for sustainable and cost-effective energy storage solutions has driven interest in biomass-derived carbon materials for supercapacitor electrodes. This study explores the valorization of coconut residue (CR), an abundant agricultural waste, as a carbon precursor for nanoporous carbon (NPC) production. NPC was synthesized via hydrothermal carbonization (HTC) of CR, followed by chemical activation using potassium hydroxide (KOH) at varying temperatures (700, 800, and 900 °C). The effects of activation temperature on the structure and electrochemical performance of the NPC were systematically investigated. The activated materials exhibited amorphous, highly porous structures, with surface areas increasing alongside activation temperature—reaching a maximum of 1969 m^2^ g^−1^ at 900 °C. Electrochemical characterization was conducted using a three-electrode setup through cyclic voltammetry (CV) and galvanostatic charge–discharge (GCD) in a 1 M Na_2_SO_4_ electrolyte. The sample activated at 900 °C with a CR:KOH weight ratio of 1:2.5 achieved the highest specific capacitance of 52 F g^−1^ at a specific current of 1 A g^−1^. These findings underscore the potential of CR as a low-cost and sustainable raw material for fabricating efficient electrode materials in energy storage applications.

## 1. Introduction

In the modern era, people place utmost importance on energy and use it for many applications and technological developments. As technology advances, the demand for energy increases accordingly. Most energy currently comes from fossil fuels and nuclear power, which are non-renewable and are depleting sources. Renewable energy sources [1,2] (wind, solar, geothermal, and bioenergy) are sustainable and exciting. However, the drawback of renewable energy is its unstable flow, relying on factors such as weather conditions, seasons, and time of day. Energy storage [3,4,5,6] devices are intermediaries to store and distribute energy. Batteries and supercapacitors are widely used energy storage devices with distinct electrochemical properties. Batteries offer high energy density for long-term storage but have low power density, limiting rapid energy delivery. In contrast, supercapacitors provide high power density for fast charge and discharge cycles but have low energy density, restricting long-term storage capacity.

Supercapacitors are gaining prominence in energy storage systems due to their high-power density, well charge–discharge capabilities, and long cycle life. Based on charge storage mechanisms, they are classified into three types: electric double-layer capacitors (EDLCs) [7], which rely on electrostatic interactions (non-Faradaic process); pseudo-capacitors (PCs) [8,9], which utilize redox reactions (Faradaic process) [10,11,12]; and hybrid supercapacitors, which combine both for enhanced performance (both Faradaic and non-Faradaic processes). Electrode materials significantly impact supercapacitor efficiency and stability. Various materials, including hierarchical porous carbons [13,14] and transition metals [15,16,17], have been explored, with carbon-based materials being the most preferred due to their low cost, high surface area, excellent conductivity, and thermal and chemical stability.

In recent years, Hui Shao et al. summarized the advancements in NPC for electrochemical double-layer capacitors in 2020, concluding that the capacitance of NPC was enhanced by a high specific surface area, pore size density below 1 nm, and the curvature of carbon within nanopores [18]. NPC materials have emerged as leading candidates for supercapacitor electrodes due to their high surface area, excellent electrical conductivity, chemical stability, and low cost. Biomass-derived carbon is particularly appealing due to its renewable nature, low cost, and environmental benefits [19,20,21,22,23]. Biomass is a type of waste that is abundantly produced daily [24,25], such as rice husk [26], mangosteen peels [27], salacca peel [28], coconut shell [29], coconut husk [30], and CR [31]. Among various biomass sources, CR, a fibrous byproduct remaining after coconut milk extraction, has received relatively limited attention compared to coconut shell and husk. However, CR is abundantly available, renewable, and often discarded as agricultural waste, posing environmental disposal issues. Unlike harder coconut derivatives, CR has a naturally porous and lignocellulosic structure, which can facilitate the development of hierarchical porosity during carbonization. These characteristics make CR an excellent candidate for producing high-surface-area carbon materials. The novelty of this study lies in the use of CR as a unique precursor to synthesize nanoporous carbon via a two-step route combining hydrothermal carbonization (HTC) and KOH activation. This approach not only valorizes a neglected waste stream but also results in a carbon material with tunable porosity, high surface area, and promising electrochemical properties. Our work introduces a sustainable pathway for converting low-value CR into functional materials for energy storage applications, expanding the utility of coconut-based biomass in supercapacitor research.

Regarding the literature, the HTC was a two-step synthesis process for converting biomass into porous carbon materials. The first step, the hydrothermal (HT) process [32,33,34], was an effective technique for converting biomass into hydrochar [35,36,37] under low-temperature, compressed water conditions. This process was prepared to achieve high carbon content and to increase the mass yield of carbon in the subsequent steps. Rebaz O. Hasan et al. [38] demonstrated the influence of temperature and reaction time on the efficiency of hydrochar synthesis through the HT process, leading to carbon content and an enhanced O/C and H/C ratio. In the second step, the carbonization process [39,40], the hydrochar was subjected to high temperatures in an inert atmosphere (such as argon [41], carbon dioxide [42], or nitrogen) to remove volatile components. High temperatures can promote the development of increased carbon content as noted by Hye-Min Lee [43]. However, the reduced yield due to mass loss and significant ash production is the downside. The internal gas flow (such as argon, carbon dioxide, or nitrogen) to create a quasi-vacuum condition can mitigate incomplete combustion and reduce ash formation. The HTC process produces NPC material after chemical activation at high temperatures. For improving porosity and surface area of carbon materials, carbonization coupled with chemical activation is a single-step process that raw materials are infused with chemical activating agents such as sodium hydroxide (NaOH), potassium carbonate (K_2_CO_3_) [44], zinc chloride (ZnCl_2_) [45], phosphoric acid (H_3_PO_4_) [46,47], sodium carbonate (Na_2_CO_3_) [34], and potassium hydroxide (KOH) [48] before being subjected to high temperatures. These chemical agents dehydrate the raw material, promote the formation of cross-links, and prevent tar formation, resulting in a higher yield of activated carbon with a higher surface area and a more extensive micropore network compared to physical activation. The report by Eka Marya Mistar et al. [49] improved the chemical activation process using KOH to enhance the development of pore diameter. The porous size was further developed into micropores [50]. Activation with KOH was a well-established method to increase the carbon material’s porosity and surface characteristics, producing NPC highly suitable for supercapacitor applications. This approach leverages biomass’s abundant, low-cost, and renewable nature, such as CR, making it an environmentally friendly and sustainable route for producing advanced materials for energy storage devices. As mentioned above, this study aimed to synthesize NPC from CR waste as a carbon source for supercapacitor electrodes. The synthesis process involved converting the waste into hydrochar through HT treatment, which served as a precursor for high carbon content. The hydrochar was chemically activated using KOH at various weight ratios (1:1.5 to 1:3 *w/w*) and carbonized at different temperatures at 700 to 900 °C for 1 h under a nitrogen gas atmosphere. The resulting product was NPC. We enhanced the physicochemical properties, including morphology, chemical and structural characteristics, surface area, and pore size, to improve its suitability and electrochemical performance as a carbon source for supercapacitor electrodes. Electrochemical analyses, including potential measurements using a three-electrode system, were conducted, and the results were presented in cyclic voltammetry (CV) and galvanostatic charge–discharge (GCD) curves.

## 2. Materials and Methods

### 2.1. Materials and Chemicals

Coconut residue (CR), obtained after extracting coconut milk, was sourced from a local market in Bangkok, Thailand. The CR was crushed and sieved to obtain particles approximately 0.45 mm in size. It was then dried at 80 °C for 24 h in an electric oven (UN55, Memmert, Schwabach, Germany) to remove moisture. Potassium hydroxide (KOH, 85%) and hydrochloric acid (HCl, 36.5%) were purchased from CARLO ERBA Reagents Co., Ltd., S.A.S (Val de Reuil, France). High-purity nitrogen gas (99.99%) used during the synthesis process was supplied by Linde (Bangkok, Thailand). Nafion^®^ DE-521 (5%, Sigma-Aldrich, St. Louis, MO, USA), isopropanol (C_3_H_8_O, 99.8%), and sodium sulfate (Na_2_SO_4_, 99.9%) were obtained from LabScan Co., Ltd. (Bangkok, Thailand), and used in the electrochemical test.

### 2.2. Synthesis of Nanoporous Carbon (NPC)

**Figure 1:**
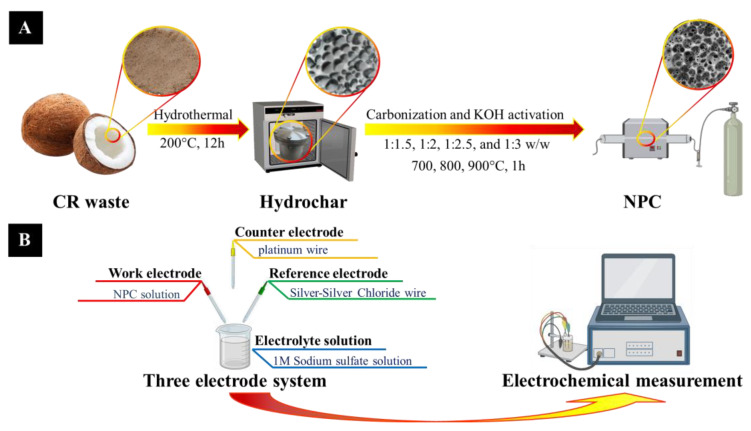
Schematic illustration depicting (**A**) the entire preparation process and (**B**) the test performance of NPC.

The synthesis of NPC from CR waste involved hydrothermal carbonization (HTC) followed by KOH activation, as illustrated in Figure 1A. Initially, the CR was mixed with deionized (DI) water in a 1:2 weight ratio and subjected to hydrothermal treatment in a Teflon-lined autoclave at 200 °C for 12 h. The resulting hydrochar was then dried at 105 °C for 24 h in an electric oven. In the subsequent carbonization and activation steps, the hydrochar was mixed with KOH at varying weight ratios (1:1.5, 1:2, 1:2.5, and 1:3) using a mortar for 15 min. The mixtures were dried again at 105 °C for 20 h. Activation was carried out at 700, 800, and 900 °C for 1 h under a nitrogen atmosphere flowing at 100 mL min^−1^, with a heating rate of 10 °C min^−1^. Post-activation, the samples were thoroughly washed with DI water and 0.1 M HCl until a neutral pH was achieved. The final drying was conducted at 105 °C for 24 h. The resulting samples were labeled according to activation temperature and KOH ratio, e.g., NPC700-K1.5, NPC800-K1.5, NPC900-K1.5, etc.

### 2.3. Characterization

Functional groups on the carbon surface were identified using Fourier-transform infrared (FTIR) spectroscopy (PerkinElmer, Waltham, MA, USA) in the range of 400–4000 cm^−1^. Crystallinity was evaluated using X-ray diffraction (XRD, Rikagu, Tokyo, Japan) with Cu Kα radiation (40 kV, 30 mA), scanned at a rate of 0.01° s^−1^ over a 2θ range of 10–80°. The structural order of the samples was further analyzed by Raman spectroscopy (Thermo Fisher Scientific, Waltham, MA, USA) using a solid-state Nd:YAG laser in the 400–4000 cm^−1^ range. Surface area and porosity were characterized by nitrogen adsorption–desorption isotherms using a Quantachrome instrument (Anton Paar, Boynton Beach, FL, USA). The Brunauer–Emmett–Teller (BET) method was used to determine the specific surface area. Total pore volume (V_total_) was derived from nitrogen adsorption at a relative pressure of 0.99. Micropore volume (V_micro_) was calculated using the t-plot method, and mesopore volume (V_meso_) was estimated by subtracting V_micro_ from V_total_.

### 2.4. Preparation of the Three-Electrode System

Electrochemical performance was evaluated using a three-electrode setup controlled by a VSP potentiostat with EC-Lab V10.33 software (Biologic, Seyssinet-Pariset, France). Cyclic voltammetry (CV) and galvanostatic charge–discharge (GCD) tests were performed, as illustrated in Figure 1B. CV was conducted in the potential window of 0–1 V (vs. Ag/AgCl) at a scan rate of 10–100 mV s^−1^. GCD measurements were carried out within the same potential range at a specific current of 1–20 A g^−1^, using 1 M Na_2_SO_4_ as the electrolyte at room temperature. The working electrode was prepared by drop-casting 3 µL of NPC dispersion onto a glassy carbon (GC) electrode (3 mm diameter, ALS Co., Ltd., Brisbane, Australia). Prior to use, the GC electrode was polished sequentially using a 0.1 µm diamond polishing pad, a 0.05 µm alumina slurry, and finally with DI water to remove any residual particles. The NPC dispersion was prepared by sonicating a mixture of 5 mg NPC, 475 µL DI water, 475 µL isopropanol, and 50 µL Nafion solution for 60 min. A platinum wire (ALS Co., Ltd., Brisbane, Australia) served as the counter electrode, and an Ag/AgCl electrode immersed in saturated KCl was used as the reference. The specific capacitance (***C_S_***) was calculated from the GCD discharge curve using the following equation:

(1) $$c_{s}=\frac{I}{m}\int \frac{1}{V\left(t\right)}dt$$

where ***I*** is the applied constant current (A), ***m*** is the mass of active material (g), and ***V***(***t***) is the potential as a function of time (s).

## 3. Results and Discussions

### 3.1. The Effect of HT

**Figure 2:**
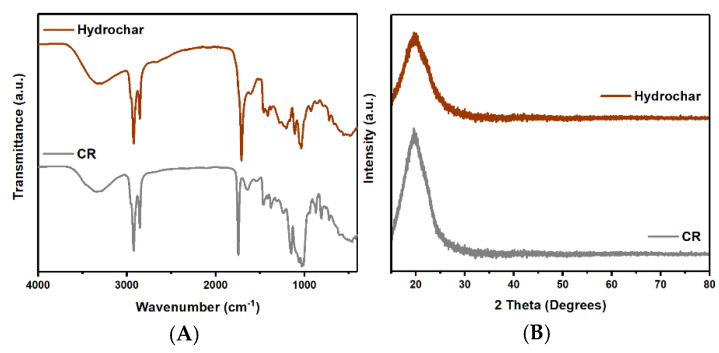
(**A**) FTIR spectra and (**B**) XRD patterns of CR and hydrochar.

**Figure 3:**
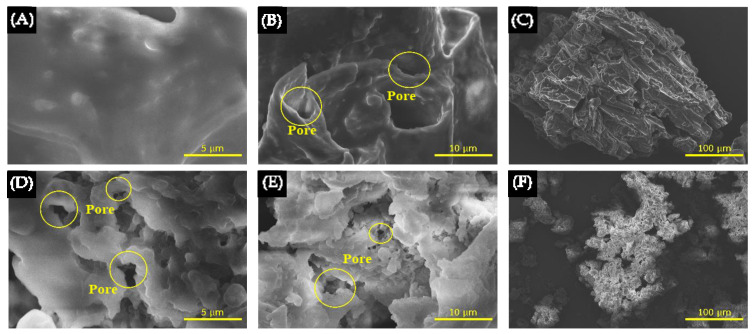
SEM morphology of CR (**A**–**C**) and hydrochar (**D**–**F**) at 20,000×, 10,000×, and 1000×, respectively.

In Figure 2A, the FTIR graph shows strong and broad peak range of 2500–3300 cm^−^^1^, corresponding to -OH stretching. A strong peak range of 1706–1720 cm^−^^1^ indicates C=O stretching, characteristic of carboxylic acid functional groups [51]. The 1020–1250 cm^−^^1^ peak shows a medium peak corresponding to C-N stretching, typical of amine groups [52]. The peak of the hydrochar range around 700–1500 cm^−^^1^ decreased, indicating the dehydration of the -OH groups [52], decomposition of the biomaterial structure, and depolymerization of the cellulose peaks when compared to hydrochar with CR waste, as displayed in Figure 2A. The findings align with the reports by Cheng Chen et al. and Khan Ahmed et al. [53,54]. In Figure 2B, the XRD patterns show sharp peaks at 2θ around 19–22°. The results were consistent with those of Zaira Zaman Chowdhury et al. and Francesco Velti et al. suggesting that this peak corresponded to the amorphous carbon phase in the cellulose structure [55,56]. Oil removal using hexane solution resulted in a greater loss of functional groups, as seen in Figure S1A. Figure S1B shows peaks at 2θ around 19–22° for hydrochar-Hex that are lower than those for hydrochar, indicating the loss of cellulose structure without the emergence of new carbon peaks. The yield of hydrochar decreased by 20 wt% (Figure S2A) due to the removal of moisture, decomposition, decomposition, and depolymerization of biomaterial structures such as cellulose, hemicellulose, and lignin [55,56]. The surface morphology of CR and hydrochar are shown in Figure 3. The surface morphology of CR at 10,000× displayed a pore-hollow tube shape, while a staircase-like rough plate was visible at lower magnification, illustrated in Figure 3B,C. However, the hydrochar exhibited the development of finer pores and a reduced pore size. These changes are attributed to modifications induced by heat, which reduced the organic content and disrupted the biomass structure, which conforms to the results reported by Eric Danso Boateng et al. and Sunday E. Elaigwu et al. [57,58,59].

### 3.2. The Effect of HTC Combined with KOH Activation

**Figure 4:**
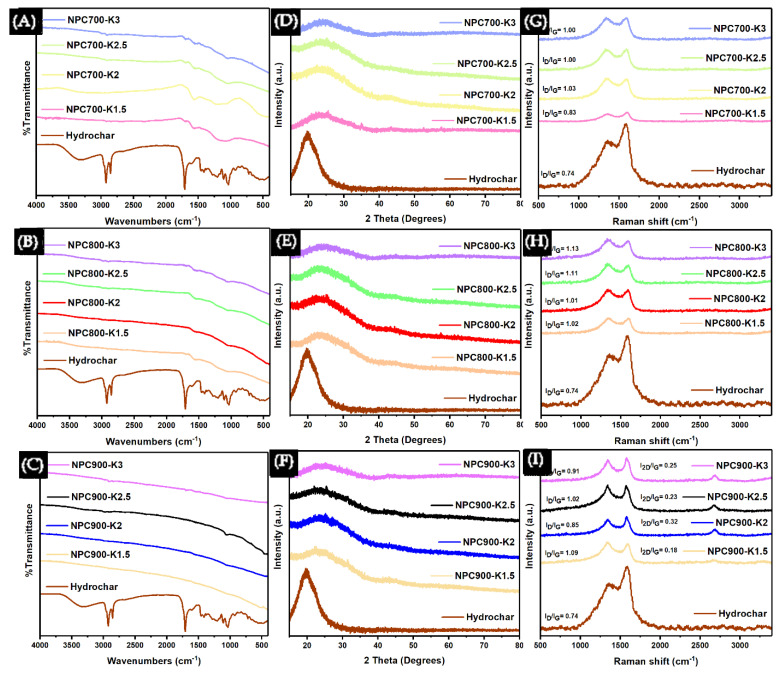
FTIR spectra of NPC at different synthesis temperatures (**A**) 700 °C, (**B**) 800 °C, and (**C**) 900 °C. XRD patterns of NPC synthesized at (**D**) 700 °C, (**E**) 800 °C, and (**F**) 900 °C. Raman spectra of NPC at (**G**) 700 °C, (**H**) 800 °C, and (**I**) 900 °C.

The chemical activation process using KOH was characterized at various ratios and temperatures to determine its effects on the material’s properties. Figure 4 shows the NPC sample after chemical activation using KOH activation. Figure 4A–C show the effects of temperature and KOH ratios on the precursor material’s functional groups. Figure 4A,B illustrate the disappearance of the -OH stretching peak at 2500–3300 cm^−1^ of carboxylic acid, as well as the reduction in the C=O stretching and C-N stretching peaks at 1706–1720 cm^−1^ and 1020–1250 cm^−1^ of amine, respectively [60]. Figure 4C shows a straight line without peaks, indicating that the functional groups of the biomaterial structure have completely decomposed at the temperature of 900 °C. Figure 4D–F show the effects of temperature on the crystalline structure of the precursor material, illustrate a decrease in the peak of around 19–22° of cellulose, and the emergence of peaks range of 23–26°, which correspond to the 002 planes of the amorphous phase of carbon [61], indicating that the sample has no significant change in the crystalline structure with varying temperatures or increasing catalyst concentration. The NPC shows the dehydration of -OH groups as indicated by the disappearance in the FTIR spectra (Figure 4A–C), decomposition with depolymerization of the biomaterial structure as evidenced by the decreased intensity (Figure 4A–C), and the emergence of carbon peaks (Figure 4D–F). Figure 4G–I illustrate the characteristics of carbon-based materials, showing defects or disordered structures (D band) and graphitization (G band) with peaks between 1350–1380 cm^−1^ and 1580–1610 cm^−1^, respectively [62]. The D and G bands are used to calculate the intensity ratios (I_D_/I_G_) to determine the structural properties of the carbon. In Figure 4G,H, the I_D_/I_G_ ratio increases or remains constant as the concentration of K increases. When the temperature increases, the I_D_/I_G_ ratio also increases, indicating that both temperature and K concentration affect the amorphous carbon structure as a monolayer (sp^2^) [62]. The highest I_D_/I_G_ value is 1.13 for NPC900-K3. Figure 4I shows that the I_D_/I_G_ ratio decreases compared to Figure 4H, but there was the appearance of the 2D band with peaks of 2600–2800 cm^−1^ [63], indicating that the formation of carbon layers is greater than one. The 2D and G bands were used to calculate the intensity ratios of the 2D band (I_2D_/I_G_). The I_D_/I_G_ ratio is inversely proportional to the I_2D_/I_G_ ratio. The I_2D_/I_G_ values were 0.18, 0.32, 0.23, and 0.25, respectively, with the highest value being 0.32 for NPC900-K2, which was less than 1, reflecting the 3D nature of graphite [64]. The yield of NPC decreased to 78–90 wt% (Figure S2B) compared to hydrochar from Table S1. Thermochemical treatment at high temperatures effectively decomposed biomaterial structures such as lignin, cellulose, and hemicellulose, enhancing the carbon structure [65]. Additionally, KOH acted as a catalyst, facilitating the degradation of these structures.

### 3.3. The Effect of NPC on Electrochemical Performance

**Figure 5:**
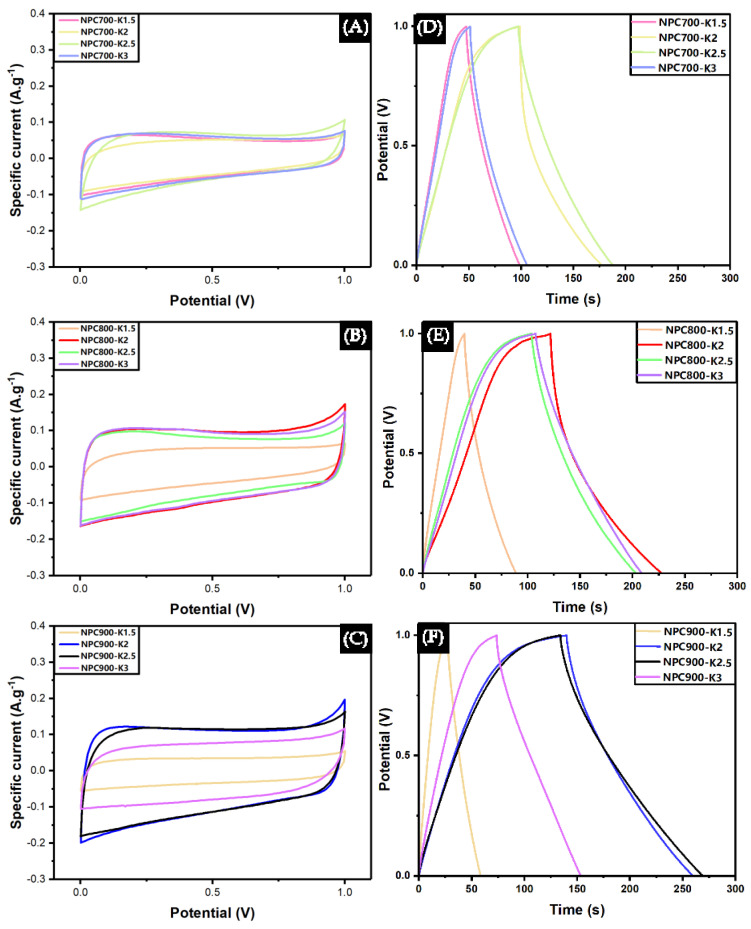
Data plots from cyclic voltammetry (CV) measurements at different temperatures (**A**) 700 °C, (**B**) 800 °C, and (**C**) 900 °C at a scan rate of 100 mV s^−1^. Galvanostatic charge–discharge (GCD) measurements at different temperatures (**D**) 700 °C, (**E**) 800 °C, and (**F**) 900 °C at a specific current of 1 A g^−1^.

The electrochemical properties of all samples were measured using a three-electrode system employing CV and GCD techniques in a 1 M Na_2_SO_4_ electrolyte solution, tested within a potential range of 0–1 V at a scan rate of 100 mV s^−1^. Figure 5A–C show the results obtained from CV measurements. Figure 5A displays sample carbonization at 700 °C, with hydrochar to KOH ratios of 1:1.5, 1:2, 1:2.5, and 1:3, respectively. The graph exhibited CV loops resembling asymmetrical rectangles, with current values ranging from −0.15 to 0.12 mA. The NPC900-K2.5 sample produced the largest CV loop. The increase in KOH increased the specific capacitance value. However, When KOH was increased beyond the optimal value, the capacitance decreased because the micropores could not be fully adsorbed and desorbed during high-speed scanning [66]. Additionally, the presence of Faradaic redox peaks was observed, consistent with FTIR results indicating remaining functional groups. Consequently, the NPC700 sample exhibited both PC and EDLC behavior. For NPC800 samples at the same ratios, the CV loops were larger than NPC700, with current values between −0.16 and 0.15 mA. The KOH ratio producing the largest CV loop was from NPC800-K2. Faradic redox was due to behaviors caused by oxygen-containing groups in porous structures [66]. However, the disappearance of Faradaic redox peaks was noted, resulting from higher calcination temperatures causing the decomposition of functional groups. Thus, the NPC800 group samples demonstrated EDLC behavior. The NPC900 group showed graph characteristics similar to those of the NPC800 group, with the highest current range from the NPC900-K2 sample at −0.2 mA to 0.19 mA. Furthermore, the CV loop was most rectangular compared to the NPC700 and NPC800 groups, indicating an excellent response typical of an ideal capacitor [67]. The effect of different scan rates at 10, 20, 50, and 100 mV s^−1^ illustrated in Figures S3–S5 show that at lower scan rates (10–20 mV s^−1^), the CV curves are more rectangular and symmetrical, reflecting rapid ion diffusion and capacitive behavior. At higher scan rates (50–100 mV s^−1^), a slight distortion appears due to increased resistive effects and reduced time for electrolyte ion migration, yet the EDLC character is retained. The GCD was employed to study the charge and discharge behavior at a specific current of 1 A g^−1^. Figure 5D shows the NPC700 group, where charge–discharge time was observed as the KOH ratio increased. The NPC700-K2 sample exhibited the longest duration. However, the resulting graph had a right-skewed triangle shape, indicating that the electrical potential increased slowly during charging and decreased rapidly during discharging. This was due to additional pseudo-capacitor behavior caused by heteroatomic oxygen [68]. Moreover, the charge–discharge times for NPC700-K2.5 and NPC700-K3 samples decreased, resulting from the collapse of the porous structure caused by the expansion of pores when using high KOH ratios. This led to a reduction in sample surface area and, consequently, decreased specific capacitance, indicating that the concentration of KOH in the mixture was unsuitable to promote the formation of the hierarchical porous carbon structure [69]. Similar graph characteristics were observed for the NPC800 and NPC900 groups at hydrochar: KOH ratios of 1:2.5 and above, as shown in Figure 5D–F. Nevertheless, the NPC800 and NPC900 groups maintained longer charge–discharge times, partly due to increased surface area as pyrolysis temperature increased. Higher calcination temperatures allowed hydrochar to react more, creating micropores and mesopores crucial for enhancing the samples’ charge storage capacity. The effect of different specific current at 1, 2, 5, 10, and 20 A g^−1^ is illustrated in Figures S3–S5. The GCD curves of all condition show nearly symmetric triangular shapes and confirm ideal EDLC behavior. The discharge times decrease with increasing current density due to the intensified IR drop and limitations in ion diffusion at higher currents. Nevertheless, both NPC700 and NPC800 maintain relatively stable performances across varying specific currents, indicating good rate capability. The NPC900-K2 with NPC900-K2.5 exhibited high capacitance at low specific currents; however, its rate performance declined slightly at higher specific currents, likely due to excessive activation at 900 °C. This may lead to pore enlargement, reduced surface wettability, and limited ion accessibility. After calculating the capacitance, as illustrated in Figure S6 samples NPC800-K2, NPC900-K2, and NPC900-K2.5 showed 38, 45, and 52 F g^−1^ capacitances, respectively.

**Figure 6:**
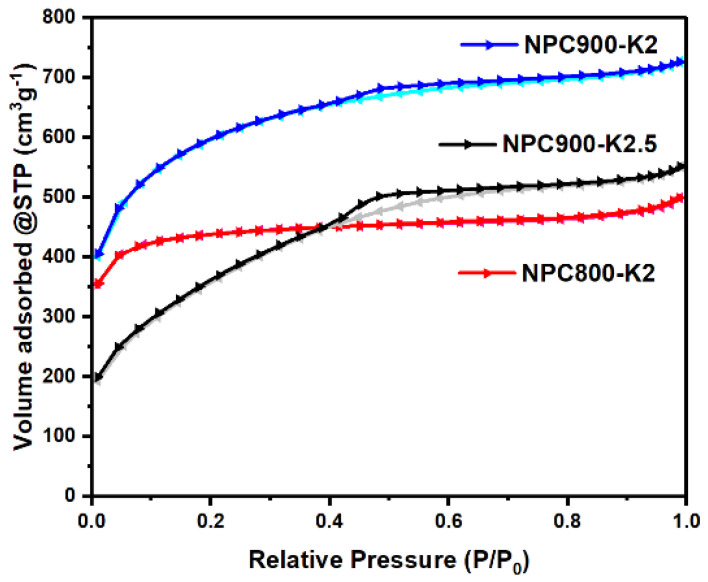
N_2_ adsorption–desorption isotherms of NPC800-K2, NPC900-K2, and NPC900-K2.5.

**Figure 7:**
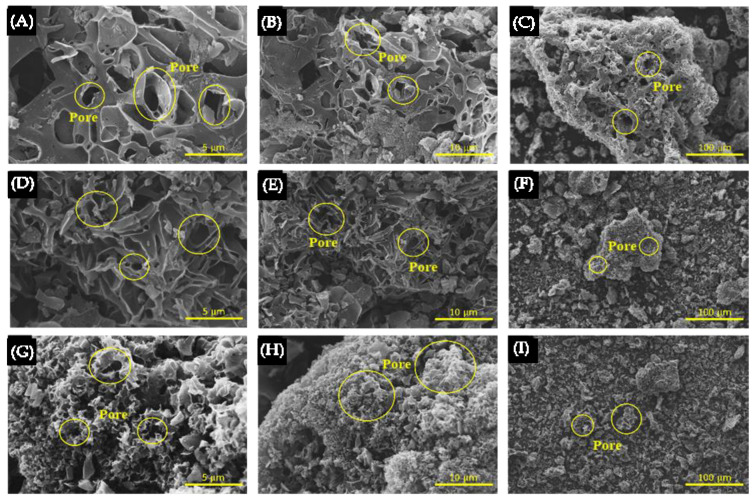
SEM morphology of NPC800-K2 (**A**–**C**), NPC900-K2 (**D**–**F**), and NPC900-K2.5 (**G**–**I**) at 20,000×, 10,000×, and 1000×, respectively.

Figure 6 presents the N2 adsorption–desorption isotherm of NPC800-K2, NPC900-K2, and NPC900-K2.5 conditions by the BET method. The low pressure (P/P0 < 0–0.4) of all samples exhibiting type I isotherm characteristics. The hysteresis loop at the high loop (P/P0 > 0.4) of the NPC900 group showed type IV isotherm, indicating that NPC900-K2 and NPC-2.5 suggest the presence of micropores and mesopores [70]. The NPC800-K2 displayed type I adsorption–desorption isotherms, which revealed the presence of many micropores. The BET results are listed in Table 1. The surface area (S_BET_) values of NPC800-K2, NPC900-K2, and NPC900-K2.5 are 1331.67, 1969.23, and 1385.51 m^2^ g^−1^, and surface macropores and mesopores (S_marco-meso_) are 271.83, 1170.66, and 1311.83 m^2^ g^−1^, respectively. It demonstrated that the micropores began to collapse and small mesopores formed when the temperature or KOH ratios were increased, as seen when comparing NPC800-K2, NPC900-K2, NPC900-K2, and NPC900-2.5. As the mesopore volume increased, the specific capacitance also increased [66]. The structural morphologies of NPC were compared based on various KOH activation ratios of 1:2 and 1:2.5 *w*/*w* and carbonization temperatures of 800 and 900 °C, as illustrated in Figure 7. The SEM images at different magnifications in Figure 7 depict the characteristic morphologic properties of NPC exhibiting amorphous size distribution, non-defined ornamentation, and a porous structure. When compared, NPC800-2 and NPC900-2 showed a significant increase in porosity at high magnification due to the thermal process, leading to the modification of the structure into highly porous carbon and disrupted pore walls through carbonization observed in Figure 7A,B,D,E. In addition, increasing the KOH activator resulted in the loss of the characteristic shape in the NPC900-2 and NPC900-2.5 samples as illustrated in Figure 7D–I. The modification from micropore to mesopore was confirmed through the results of BET analysis. These findings suggested chemical activation with KOH, consistent with previous reports by Min Zhang and Yanyan Fang [71,72].

## 4. Conclusions

In this study, nanoporous carbon (NPC) was successfully synthesized from coconut residue (CR) waste via hydrothermal carbonization (HTC) followed by KOH activation at temperatures ranging from 700 to 900 °C. The hydrochar derived from CR demonstrated functional groups indicative of carbon-rich precursors suitable for further carbonization. Following activation, the resulting NPC materials showed significant structural transformations, including the cleavage of functional groups, the formation of amorphous carbon phases, and the emergence of multilayered graphite structures. Thermal treatment and increased KOH ratios notably enhanced porosity development, yielding high surface areas dominated by micropores and mesopores. The highest BET surface area (S_BET_) achieved was 1969 m^2^ g^−1^ for the NPC900-K2 sample. Electrochemical performance, evaluated using a three-electrode system in a 1 M Na_2_SO_4_ electrolyte, revealed that the sample NPC900-K2.5, with the highest S_macro-meso_ value of 1311.83 m^2^ g^−1^, exhibited the highest specific capacitance of 52 F g^−1^ at a specific current of 1 A g^−1^. These results underscore the importance of mesopores in facilitating efficient charge storage at high current densities. Furthermore, the findings suggest that improving the microporous structure may mitigate the loss of capacitance at such conditions. Overall, the synthesized NPC from CR waste exhibits promising characteristics for application in electrochemical energy storage devices and holds potential for future commercial development.

## Figures and Tables

**Table 1:** Textural parameters obtained from N_2_ adsorption–desorption isotherms of NPC800-K2, NPC900-K2, and NPC900-K2.5.

Condition	S_BET_ (m^2^/g)	S_micro_ (m^2^/g)	S_marco-meso_ (m^2^/g)	V_total_ (cm^3^/g)	V_micro_ (cm^3^/g)	D_average_ (nm)
NPC800-K2	1331.67	1059.84	271.83	0.75	0.56	37.39
NPC900-K2	1969.23	798.57	1170.66	1.11	0.41	29.32
NPC900-K2.5	1385.51	73.68	1311.83	0.83	0.02	29.55

## Data Availability

The data presented in this study are available on request from the corresponding author. The data are not publicly available due to institutional restrictions.

## Supplementary Materials

The following supporting information can be downloaded at https://www.mdpi.com/article/10.3390/polym17131752/s1, Figure S1: (A) FTIR spectra and (B) XRD patterns comparing oil removal from coconut residue and hydrochar using a hexane solution; Table S1. Mass yield of NPC temperature at 700, 800, and 900 °C for KOH ratios at 1.5, 2, 2.5, and 3; Figure S2. Mass yield of (A) hydrochar compared to oil removal using a hexane solution and (B) NPC under all conditions; Figure S3. CV and GCD curves of NPC700 difference KOH ratios at (A, B) K1.5, (C, D) K2, (E, F) K2.5, and (G, H) K3. CV at scan rates of 10–100 mV s^−1^; GCD at specific current of 1–20 A g^−1^; Figure S4. CV and GCD curves of NPC800 difference KOH ratios at (A, B) K1.5, (C, D) K2, (E, F) K2.5, and (G, H) K3. CV at scan rates of 10–100 mV s^−1^; GCD at specific current of 1–20 A g^−1^; Figure S5. CV and GCD curves of NPC900 difference KOH ratios at (A, B) K1.5, (C, D) K2, (E, F) K2.5, and (G, H) K3. CV at scan rates of 10–100 mV s^−1^; GCD at specific current of 1–20 A g^−1^; Figure S6. The Specific capacitance (Cs) value (A) graph and (B) table of GCD graph of NPC all conditions.
