# Supplementary material for: Coconut Residue-Derived Nanoporous Carbon via Hydrothermal Carbonization for Nanoporous Carbon-Based Supercapacitor Electrodes
Source: Polymers (Basel). 2025 Jun 25;17(13):1752. doi: 10.3390/polym17131752 (PMC12251755; doi:10.3390/polym17131752)
Supplement: Supplementary file 1 [file polymers-17-01752-s001.zip › polymers-3645997-supplementary.pdf]

Coconut Residue-Derived Nanoporous Carbon via Hydrothermal Carbonization for Nanoporous Carbon-Based Supercapacitor Electrodes

Kemchat Ruenroengrit [1], Jumpon Kunyuan [2], Nuttapong Ruttanadech [2], Napat Kaewtrakulchai [3], Pramote Puengjinda [4], Nattapat Chaiammart [5], Sutee Chutipaijit [1], Achanai Buasri [5], Masayoshi Fuji [6], Apiluck Eiad-Ua [1,*] and Gasidit Panomsuwan [7]

[1] Nanoscience and Nanotechnology, College of Materials Innovation and Technology, King Mongkut's Institute of Technology Ladkrabang, Ladkrabang, Bangkok 10520, Thailand; 65116002@kmitl.ac.th (K.R.); sutee.ch@kmitl.ac.th (S.C.)

[2] King Mongkut's Institute of Technology Ladkrabang Prince of Chumphon Campus, Chumphon 86160, Thailand; jumpon.ku@kmitl.ac.th (J.K.); nuttapong.ru@kmitl.ac.th (N.R.)

[3] Kasetsart Agricultural and Agro-Industrial Product Improvement Institute, Kasetsart University, Bangkok 10900, Thailand; knapat.kara@gmail.com

[4] Deutsche Gesellschaft für Internationale Zusammenarbeit (GIZ) GmbH, Bangkok 10110, Thailand; pramote.puengjinda@giz.de

[5] Department of Materials Science and Engineering, Faculty of Engineering and Industrial Technology, Silpakorn University, Nakhon Pathom 73000, Thailand; nattaphat.c@ku.th (N.C.); achanai130@gmail.com (A.B.)

[6] Advanced Ceramic Center, Nagoya Institute of Technology, Gifu 466-8555, Japan; fuji@nitech.ac.jp

[7] Department of Materials Engineering, Faculty of Engineering, Kasetsart University, Chatuchak, Bangkok 10900, Thailand; gasidit.p@ku.ac.th

* To whom correspondence should be addressed.

Tel.: +66–2–329–8300 ext. 3132; Fax: +66–2–329–8625, E-mail: apiluck.ei@kmitl.ac.th

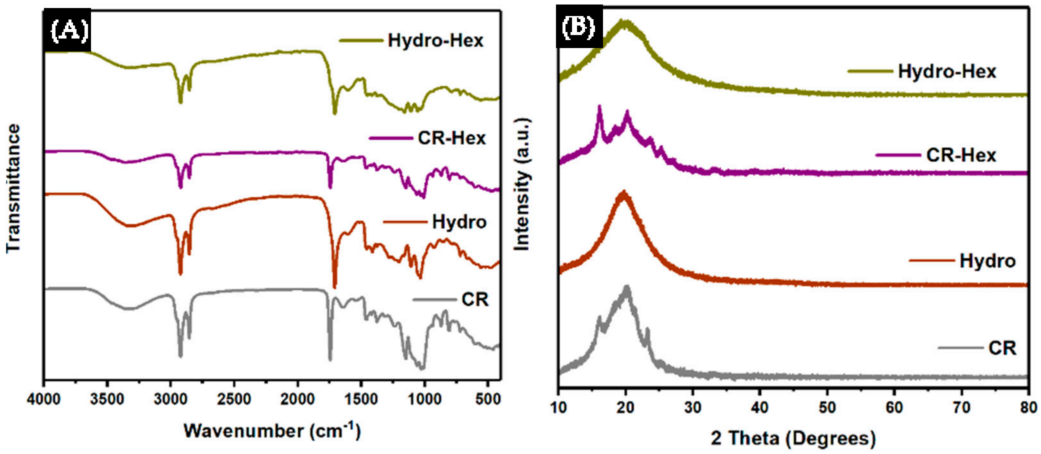

Figure S1. (A) FTIR spectra and (B) XRD patterns comparing oil removal from coconut residue and hydrochar using a hexane solution.

Table S1. Mass yield of NPC temperature at 700, 800, and 900 °C for KOH ratios at 1.5, 2, 2.5, and 3.

Condition	Mass Yield (%)
Hydrochar	80.12
NPC700-K1.5	21.54
NPC700-K2	20.40
NPC700-K2.5	20.25
NPC700-K3	20.12
NPC800-K1.5	16.23
NPC800-K2	15.89
NPC800-K2.5	15.56
NPC800-K3	15.42
NPC900-K1.5	10.87
NPC900-K2	10.52
NPC900-K2.5	10.32
NPC900-K3	10.22

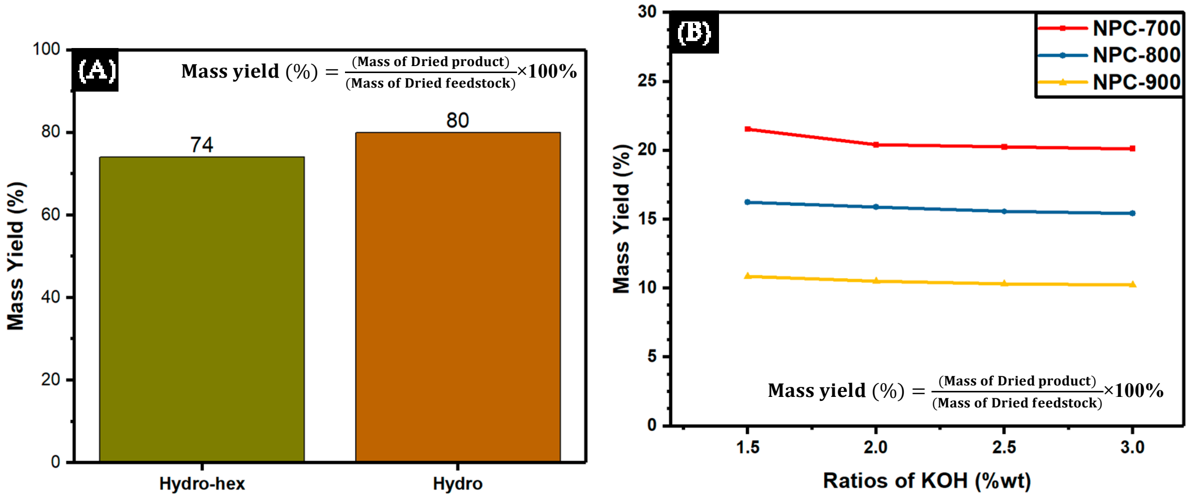

Figure S2. Mass yield of (A) hydrochar compared to oil removal using a hexane solution and (B) NPC under all conditions.

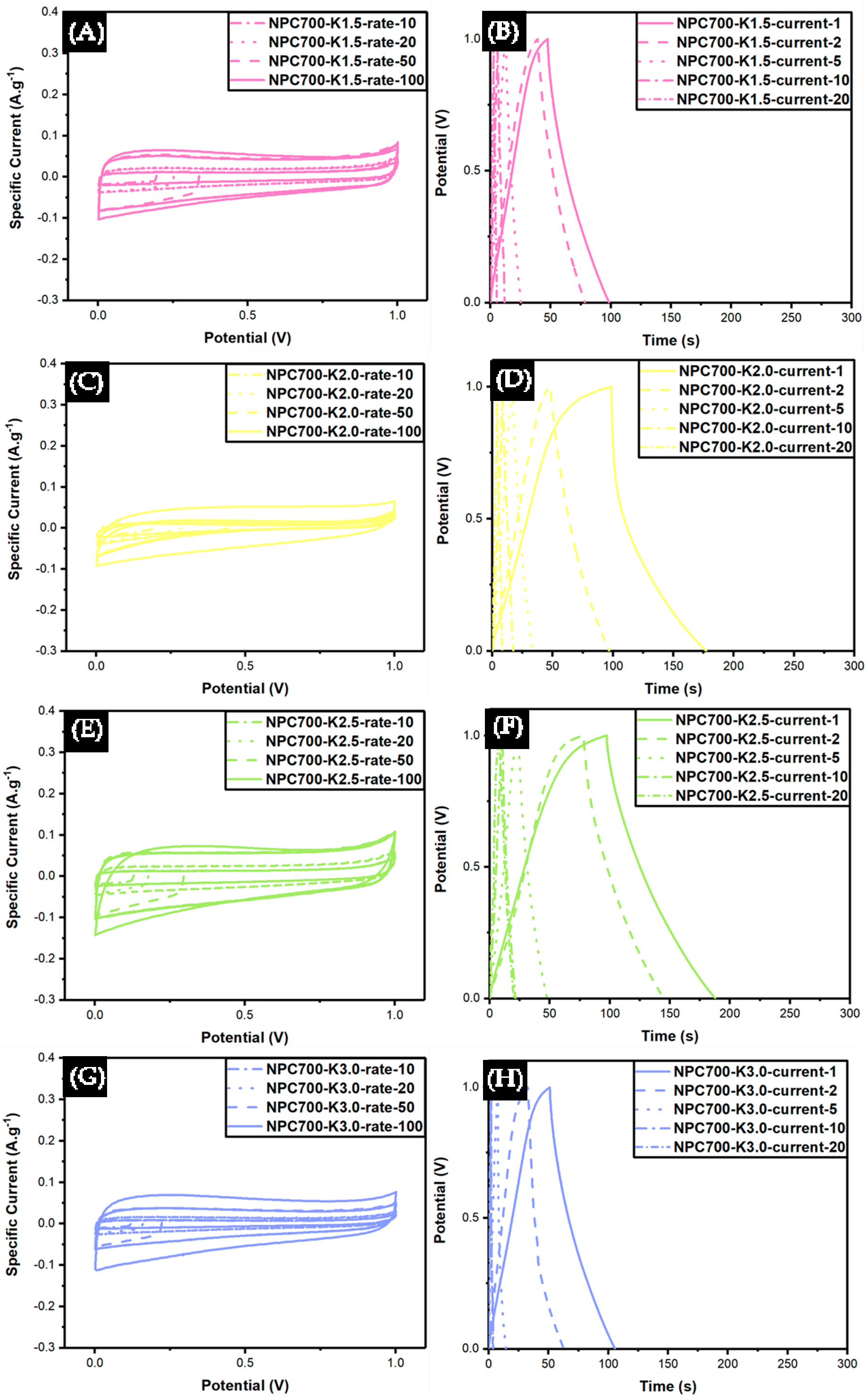

Figure S3. CV and GCD curves of NPC700 difference KOH ratios at (A, B) K1.5, (C, D) K2, (E, F) K2.5, and (G, H) K3. CV at scan rates of 10–100 mV s^{-1}; GCD at specific current of 1–20 A g^{-1}.

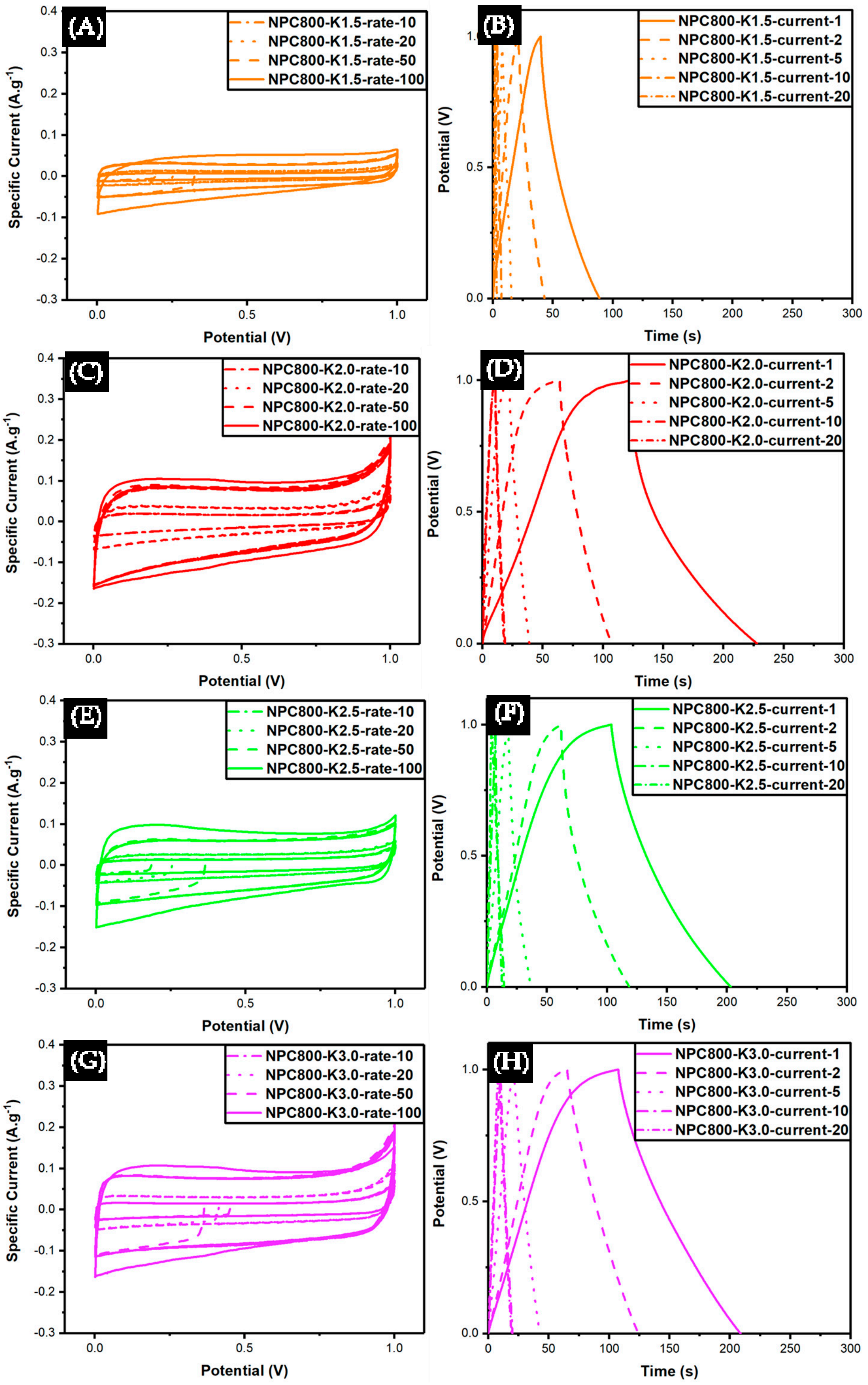

Figure S4. CV and GCD curves of NPC800 difference KOH ratios at (A, B) K1.5, (C, D) K2, (E, F) K2.5, and (G, H) K3. CV at scan rates of 10–100 mV s^{-1}; GCD at specific current of 1–20 A g^{-1}.

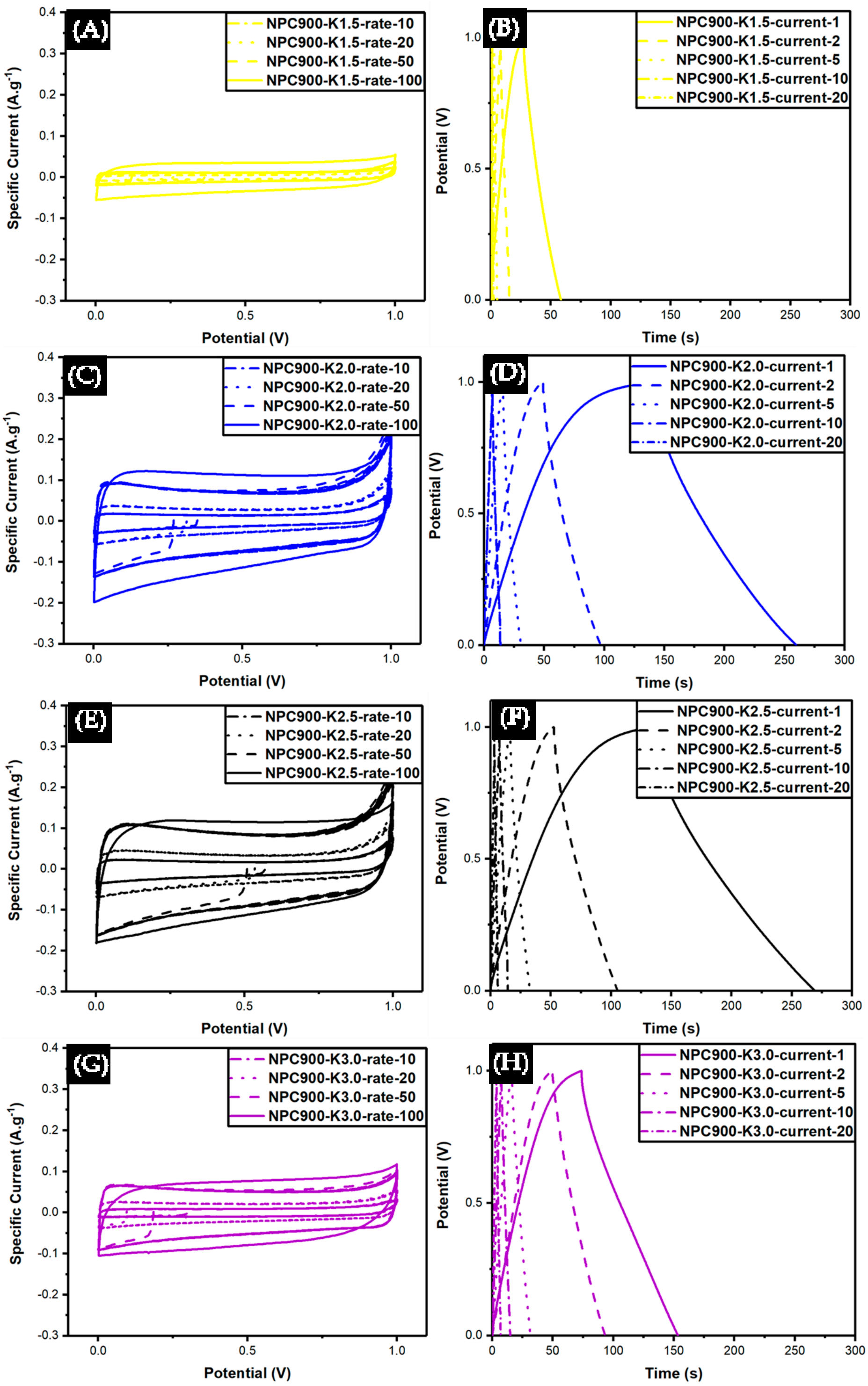

Figure S5. CV and GCD curves of NPC900 difference KOH ratios at (A, B) K1.5, (C, D) K2, (E, F) K2.5, and (G, H) K3. CV at scan rates of 10–100 mV s^{-1}; GCD at specific current of 1–20 A g^{-1}.

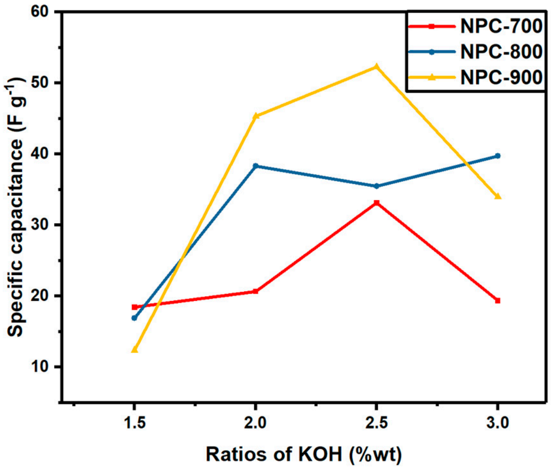

Condition	Specific capacitance of GCD graph (F g^{-1})
NPC700-K1.5	18.41
NPC800-K1.5	16.91
NPC900-K1.5	12.38
NPC700-K2	20.64
NPC800-K2	38.33
NPC900-K2	45.32
NPC700-K2.5	33.11
NPC800-K2.5	35.46
NPC900-K2.5	52.28
NPC700-K3	19.35
NPC800-K3	39.75
NPC900-K3	33.95

Figure S6. The Specific capacitance (Cs) value (A) graph and (B) table of GCD graph of NPC all conditions.
